# An Improved QSPR Modeling of Hydrocarbon Dipole Moments

**DOI:** 10.1100/tsw.2004.193

**Published:** 2004-11-18

**Authors:** Igor V. Nesterov, Andrey A. Toropov, Pablo R. Duchowicz, Eduardo A. Castro

**Affiliations:** ^1^ Scientifical Research Institute Algorithm-Engineering, F. Khodjaev Street 34, 700025 Tashkent, Uzbekistan; ^2^ INIFTA, Departamento de Química, Facultad de Ciencias Exactas, Universidad Nacional de La Plata, Diagonal 113 y 64, Sucursal 4, C. C. 16, La Plata 1900, Argentina

**Keywords:** dipole moments, hydrocarbons, QSPR, topological descriptors, molecular connectivity method, multivariable regression analysis, E-State Indices

## Abstract

Dipole moments of hydrocarbons are not an easy property to model with conventional 2D descriptors. A comparison of the performance of the most commonly used sets of topological descriptors is presented, each set containing descriptors derived from the regular and Detour distance matrix, Electrotopological State Indices, and the basic number of atoms of each type and bonds. Data were taken on a representative set of 35 hydrocarbon dipole moments previously reported and the classical multivariable regression analysis for establishing the models is employed.

## INTRODUCTION

The permanent electric dipole moment (DM) is an important physical chemistry property. By definition, the electric dipole moment vector points from the center of the positive charge distribution to the center of the negative charge distribution. It furnishes valuable information about the structure and polarity of the molecule under consideration. Evidently, the necessary condition of a suitable theoretical method is its capability to reproduce the experimental dipole moment.

Among the theoretical methods that are employed with significant success in the field of prediction of molecular physical chemistry properties and/or biological activities, the mathematical procedures based on topological concepts are very important. They are normally known as Quantitative Structure-Property/Activity Relationships (QSPR/QSAR). These methods are mainly based on nonempirical, structure-related graph theoretical molecular descriptors, where molecules are considered as a set of vertices attached to each other by a set of nonmetrical connections. These computational procedures allow one to forecast properties or activities of specific chemical compounds once the properties or activities of a set of those compounds has been modeled[1,2,3,4,5,6,7].

A rather successful mathematical topological method employed in QSPR/QSAR studies is the molecular connectivity method that permits one to derive a set of graph-theoretical indices of wide applicability by means of a well-defined set of rules[8,9,10,11,12,13,14]. The use of the so-called "pure" topological indices, i.e., those that have information only about the relationships of connectivity between atoms or bonds in the molecules, has proved to be quite simple and direct and they provide a suitable tool for accurate predictions of properties and activities. Classical examples of these indices are the Randic branching index[8], Hosoya Z index[15], Balaban J index[16], the Wiener index[17], and bond connectivity index[18].

In a recent paper[19], we presented some results for dipolar moments via QSPR modeling by means of a flexible set of molecular descriptors, comparing the predictions with available experimental and other theoretical results in order to extend the study of this physical chemistry property and, at the same time, to analyze the capabilities of this sort of molecular indices. Although final results revealed an improvement with respect to previous data, statistical parameters associated to the regression equations were not totally satisfactory, so that we have deemed it convenient to try the analysis via some pure topological indices.

It is our purpose to analyze how the different types of topological descriptors perform for the modeling of the 35 hydrocarbon dipole moments set. This article is organized as follows: next section deals with the definition of the topological indices and then we describe the calculation procedure. The presentation of results is accompanied with the comparison with other similar data and the discussion of them. Finally, we state the main conclusions derived from the present analysis.

## TOPOLOGICAL INDICES

The two most important matrices that delineate the labeled chemical graph are the adjacency (**A**) and the distance (**D**) matrices[8,20,21]. In A, $A_{ij} = 1$ if vertices $i$ and $j$ are adjacent, 0 otherwise. $D_{ij} = l_{ij}$ if $i = j$, 0 otherwise. $l_{ij}$ is the shortest edge count between vertices $i$ and $j$[15]. The $\mathbf{D}^{-2}$ matrix is the matrix whose elements are the squares of the reciprocal distances $D_{ij}^{-2}$[22], with $i,j = 1, 2, \ldots$.

The molecular topological index (MTI), also called the Schultz index after its originator[23,24], is a molecular descriptor with structural significance and it has a number of attractive features[25,26,27]. Originally, it was defined for hydrocarbon molecules[23], but later on it was generalized for heterosystems[28]. The extended version of the MTI was tested in QSPR modeling of boiling points of alkyl alcohols and it showed to be a topological index worthy of future studies about its properties and applicability in the structure property/activity relationships.

The Schultz MTI is defined as

$$\mathrm{MTI} = \sum_{i=1}^{N} e_i \qquad (1)$$

where $e_i$ (i = 1,2, ....., N) are the elements of the following row matrix

$$v \,.\, [A + D] = [e_1\, e_2 \,.....\, e_N] \qquad (2)$$

where v is the valence (1 × N) matrix of a structure G, A is the adjacency (N × N) matrix, and D is the distance (N × N) matrix[29,30,31]. The entries in the valence row matrix v are the graph-theoretical valences.

The MTI may be extended to heterosystems in a straightforward way by substituting the elements of the A and D matrices corresponding to heteroatoms and heterobonds with the values taking into account the corrections due to the changes originated in the replacement of a carbon atom by a heteroatom.

The connectivity index $\chi = \chi(G)$ of a structure G is defined as[8]:

$$\chi = \sum_{i,j} [v_i.v_j]^{-1/2} \quad (3)$$

where $v_i$ is the valence of a vertex i in G. For heterosystems, the connectivity index is given in terms of the valence delta values $\delta^v(i)$ and $\delta^v(j)$ of atoms i and j, and it is written as $\chi^v$. This connectivity index is called the valence connectivity index and it is defined as[9,32,33]:

$$\chi^v = \sum_{i,j} [\delta^v(i)\ \delta^v(j)]^{-1/2} \quad (4)$$

where

$$\delta^v(i) = (Z_i^v - H_i)/(Z_i - Z_i^v - 1) \quad (5)$$

with $Z_i$ the atomic number of atom i, $Z_i^v$ the number of valence electrons of atom i, and $H_i$ is the number of hydrogen atoms bonded to atom i.

The Wiener W number is defined as[34]:

$$W = 1/2 \sum_i \sum_j l_{ij} \quad (6)$$

and it is the simplest first-generation topological index.

The Harary index H is based on the concept of reciprocal distance and is defined, in parallel to the Wiener index, as the half sum of the off-diagonal elements of the reciprocal molecular distance matrix $D_{ij}^{-1}$[35]:

$$H = 1/2 \sum_i \sum_j 1/l_{ij} \quad (7)$$

It should be noted that diagonal elements $D_{ii}^{-1}$ are all equal to zero by definition.

The Balaban index J (average distance connectivity index) is based on distasums[36], in order to reduce the degeneracy of previous indices, because distasums (unlike vertex degrees) are seldom identical for nonequivalent vertices in nonisomorphic graphics. The definition involves a sum of “weighted average topological distances” for all edges i-j

$$J = [q/(\mu+1)] \sum_{i\text{-}j} (l_i\ l_j)^{-1/2} \quad (8)$$

The numbers q of graph edges and μ of cycles in the graph are introduced into the above formula in order to avoid the automatic increase of J with graph size and cyclicity[37].

Kier et al.[38] have generalized the definition[3] for longer paths than edges (which can be considered as paths of length L = 1 in the graph). The three carbon atoms in a propane graph form a path of length L = 2, an *n*-butane graph is a path of length L = 3, and so on. An isobutene graph is a cluster on four vertices. Thus. We have a sum of “weighted paths”, yielding connectivity indices of higher order L:

$$^L\chi = \sum_{ijk} \{v_i\ vj\ v_{jk}\ \ldots\ldots\ldots\}^{1/2} \quad (9)$$

Two other first-generation topological indices, advocated by the Zagreb group[40] are the sum of squares of vertex degrees, and the sum of products over all edges i-j

$$M_1 = \sum_i v_i^2 \quad (10)$$

$$M_2 = \sum_{i,j} v_i . v_j \quad (11)$$

The first-, second- and third-order molecular valence connectivity indices ${}^L\chi_v$ (L = 0, 1, 2) are defined in analogy with [9] and $M_{lv}$ (l = 1, 2). Zagreb group indices based on valence vertex degrees are also defined on the basis of Eqs. 10 and 11.

Finally, $W_{max}$, $H_{max}$, $J_{max}$, and $MTI_{max}$ are analogous to those defined by Eqs. 1 and Eqs. 6–8 by resorting to the Detour matrix[41] instead of the regular distance matrix.

A particularly simple set of topological descriptors are the number of atoms of each kind and the number of classical chemical bonds. In spite of the naïveté of these descriptors, they have shown to be very valuable for this sort of calculation[42,43,44,45], so that we have deemed it suitable to employ them here.

As conventional topological descriptors characterize the molecule as a whole, i.e., molecular size or shape, it may be fruitful to employ atom-type topological indices. These are expected to reflect the role of each atom type or group in a molecule, then depicting more accurately their intermolecular interactions influencing the physical property of a molecule. This interaction between groups can be of the Van der Waals or the hydrogen bonding type. One kind of such index is the so-called Electrotopological State Index ($S_i$), based on the electronic state of each atom type and its topological nature in molecular graph, introduced by Kier et al. and used successfully in a variety of QSAR/QSPR studies of complex molecules[46,47,48,49,50,51].

The $S_i$ for atom i is defined from its Intrinsic State $I_i$ plus a perturbation term in order to take account for the molecular environment of that atom:

$$S_i = I_i + \sum_j \Delta I_{ij} \quad (12)$$

$$\Delta I_{ij} = [I_i - I_j]/ r_{ij}^m \text{ , with m = 2 generally} \quad (13)$$

$$I_i = [(2/N)^2 . \delta^v(i) - 1]/\delta(i) \quad (14)$$

with N being the principal quantum number, δ(i) the delta value for atom i:

$$\delta(i) = (\sigma_i . Z_i^v - H_i)/(Z_i - Z_i^v - 1) \quad (15)$$

and with $\sigma_i$ equal to the count of electrons in sigma orbitals. It is worthwhile to cite three recently published books in topological indices, which are rather valuable source of significant information on this issue[52,53,54].

## CALCULATION PROCEDURE

The QSPR models were built via multilinear regression analysis based on first-, second-, and third-order polynomials. The use of higher-order polynomials fitting equations is based on the fact they usually improve the prediction of physical chemistry properties or/and biological activities.

We try to minimize the functional standard deviation, defined as follows:

$$S^2 = \frac{1}{N-n-1} \sum_{j=1}^{N} r_j^2 \quad (16)$$

where $r_j$ are the residuals.

The best model for a given set of descriptors according to the criterion of smallest $S$ can be obtained by trying all the combinations of $k$ descriptors out of $n$ for $k = 1,2,...,n$. This procedure is time consuming (especially in the case of exact rational calculations) because the total number of calculations is $2^n – 1$. For that reason, we resorted to a different strategy: first we perform a linear regression with all the descriptors and remove the one with the greatest relative error $\Delta c_j/c_j$. Then, we repeat the calculation with the remaining $n – 1$ descriptors, and again we remove the descriptor with the largest relative error. We proceed exactly in the same way for $n – 2, n – 3, ...$ remaining descriptors until we have just one descriptor and the constant. Then, one has to choose the set with the minimum $S$-value. This procedure, whose denomination is due to R. Maronna, requires only $n$ calculations and enables us to single out an optimum model that is very close to or in complete agreement with that one obtained by means of the thorough search mentioned before.

Since the set of molecules under study is rather small, we need to assess the predictive power of the models through some kind of validation technique. We use the “leave-one-out” cross-validation[55].

In the present paper, the quality of the models is selected by the following statistic parameters: $R_{cal}$, $S_{cal}$, and F; the correlation coefficient; standard deviation; and Fisher test of the calibration set, respectively; absdv is the mean absolute deviation of the model; Rlou and Slou stand for the correlation coefficient and standard deviation of the leave-one-out cross-validation method.

We chose the models by first applying Maronna’s Method and then selecting the generated model that had similar values for $S_{cal}$ and $S_{lou}$. That is to say, we searched for the approximate solution near Maronna’s minimum and not when $S$ is minimum. The method is justified since the purpose of the QSAR-QSPR theories is to look for a model that can fit both the calibration data set and the validation data set with the same accuracy. One of the possible advantages of doing this is that the number of descriptors for the model usually decreases, but with a little increase in $S_{cal}$ and decrease in $R_{cal}$.

In order to avoid round-off errors in our linear-regression calculations, we resorted to computer algebra system DERIVE[56] that enables one to solve the least-square equations in exact rational mode if necessary. Although this kind of calculation is commonly slow, it is sufficiently fast for our present purposes.

## RESULTS AND DISCUSSION

First, we used a topological indices set composed of the following 18 descriptors: {W, H, J, MTI, $W_{max}$, $H_{max}$, $J_{max}$, $MTI_{max}$, ${}^0X$, ${}^1X$, ${}^2X$, $M_1$, $M_2$, ${}^0X_v$, ${}^1X_v$, ${}^2X_v$, $M_{1v}$, $M_{2v}$}. Since the initial set of descriptors is large, we resorted to a first-order model. From this set of descriptors the best model found with Maronna’s method contains 9 descriptors:

**Model 1**

$$DM = 0.0167W – 0.0853H – 0.0044MTI + 0.0002MTI_{max} – 0.0988\,{}^2X – 0.1650M_1 + + 0.2448M_2 + 0.1241M_{1v} – 0.1349M_{2v} + 0.0023$$

$R_{cal} = 0.9074$, $S_{cal} = 12.9502$, $F = 0.1030$, absdv = 0.0661, $R_{lou} = 0.8343$, $S_{lou} = 0.1164$

Within the realm of this model, there are no-outliers with absolute deviation exceeding 2.5*S. The predicted values for the property are described in Table 1. As can be seen, model 1 does not have enough predictive ability, which means that the topological indices derived from the regular and Detour distance matrix are not suitable enough for modelling dipole moments of hydrocarbons.

**TABLE 1**
**Experimental and Theoretical DM (Debye) for 35 Hydrocarbons[a]**

| ID | Molecule | $DM_{exp}$ | DM ref.[19] | DM model 1 | DM model 2 |
|---|---|---|---|---|---|
| 1 | Propyne | 0.75 | 0.777 (–0.027) | 0.851 (–0.101) | 0.724 (0.026) |
| 2 | Cyclopropene | 0.455 | 0.292 (0.163) | 0.455 (0.000) | 0.452 (0.003) |
| 3 | 1-Butene | 0.34 | 0.411 (–0.071) | 0.254 (0.086) | 0.454 (–0.114) |
| 4 | 1-Butyne | 0.80 | 0.711 (0.089) | 0.665 (0.135) | 0.731 (0.069) |
| 5 | 1,2-Butadiene | 0.401 | 0.332 (0.069) | 0.530 (–0.129) | 0.318 (0.083) |
| 6 | 2-Methylpropene | 0.503 | 0.482 (0.021) | 0.421 (0.082) | 0.449 (0.054) |
| 7 | 1,3-Cyclopentadiene | 0.416 | 0.315 (0.101) | 0.439 (–0.023) | 0.366 (0.050) |
| 8 | 2-Methylbutane | 0.13 | 0.129 (0.001) | 0.304 (–0.174) | 0.158 (–0.028) |
| 9 | 2-Methyl-1,3-butadiene | 0.38 | 0.381 (–0.001) | 0.381 (–0.001) | 0.361 (0.019) |
| 10 | 1,3-Cyclohexadiene | 0.44 | 0.511 (–0.071) | 0.474 (–0.034) | 0.432 (0.008) |
| 11 | 3,3-Dimethyl-1-butyne | 0.66 | 0.638 (0.022) | 0.651 (0.009) | 0.770 (–0.110) |
| 12 | 1,3,5-Cycloheptatriene | 0.25 | 0.338 (–0.088) | 0.385 (–0.135) | 0.356 (–0.106) |
| 13 | Styrene | 0.095 | 0.185 (–0.090) | 0.266 (–0.171) | 0.101 (–0.006) |
| 14 | Ethylbenzene | 0.59 | 0.490 (0.100) | 0.351 (0.239) | 0.506 (0.084) |
| 15 | o-Xylene | 0.62 | 0.577 (0.043) | 0.573 (0.047) | 0.501 (0.119) |
| 16 | 1,3,5,7-Nonatetraene | 0.423 | 0.349 (0.074) | 0.390 (0.033) | 0.386 (0.037) |
| 17 | 1,3,5,7,9-Undecapentaene | 0.422 | 0.372 (0.050) | 0.415 (0.007) | 0.435 (–0.013) |
| 18 | 1-Methylnaphthalene | 0.28 | 0.401 (–0.121) | 0.402 (–0.122) | 0.407 (–0.127) |
| 19 | 2-Methylnaphthalene | 0.44 | 0.401 (0.039) | 0.326 (0.114) | 0.407 (0.033) |
| 20 | 1,3,5,7,9,11-Tridecahexaene | 0.47 | 0.395 (0.075) | 0.442 (0.028) | 0.466 (0.004) |
| 21 | Fluorene | 0.28 | 0.213 (0.067) | 0.220 (0.060) | 0.280 (0.000) |
| 22 | 1,3,5,7,9,11,13-Pentadecaheptaene | 0.452 | 0.418 (0.034) | 0.476 (–0.024) | 0.451 (0.001) |
| 23 | Benzo[a]anthracene | 0.045 | 0.265 (–0.220) | –0.0043 (0.049) | –0.001 (0.046) |
| 24 | Benzo[b]fluoranthene | 0.30 | 0.284 (0.016) | 0.364 (–0.064) | 0.158 (0.142) |
| 25 | Indeno[1,2,3cd]pyrene | 0.466 | 0.302 (0.164) | 0.403 (0.063) | 0.470 (–0.004) |
| 26 | Propene | 0.36 | 0.280 (0.080) | 0.306 (0.054) | 0.447 (–0.087) |
| 27 | 2-Methylpropane | 0.132 | 0.063 (0.069) | 0.123 (0.009) | 0.114 (0.018) |
| 28 | 1-Pentyne | 0.81 | 0.777 (0.033) | 0.712 (0.098) | 0.775 (0.035) |
| 29 | Cyclohexene | 0.61 | 0.621 (–0.011) | 0.581 (0.029) | 0.568 (0.042) |
| 30 | 1-Hexyne | 0.83 | 0.843 (–0.013) | 0.843 (–0.013) | 0.840 (–0.010) |
| 31 | 1,3,5-Heptatriene | 0.355 | 0.326 (0.029) | 0.370 (–0.015) | 0.351 (0.004) |
| 32 | Toluene | 0.37 | 0.360 (0.010) | 0.348 (0.022) | 0.439 (–0.069) |
| 33 | m-Xylene | 0.37 | 0.577 (–0.207) | 0.446 (–0.076) | 0.399 (–0.029) |
| 34 | Benzo[a]pyrene | 0.037 | 0.284 (–0.247) | 0.086 (–0.049) | 0.158 (–0.121) |
| 35 | Benzo[k]fluoranthene | 0.12 | 0.284 (–0.164) | 0.139 (–0.019) | 0.158 (–0.038) |
| Absolute average deviation[b] | | — | 0.077 | 0.066 | 0.050 |

[a]Deviation between parenthesis; [b]absolute average deviation = $\sum_{j=1} \{DM_{exp}(j)\text{-}DM_{calc}(j)\}/35$.

A next step for improving the description of the property's space could be achieved by including the electronic aspects together with the topological description of the molecular structure. This would be well accomplished with the $S_i$'s. The predictor variables were calculated for each i fragment of the molecule by taking the mean of its $S_i$ values. However, the resulting linear, quadratic, and cubic models with the set of $S_i$ descriptors were of inferior quality in comparison to the statistic parameters of model 1. Including no-linealities such as cross-terms in the original $S_i$'s did not cause any remarkable effects.

Then we have tried a set containing the basic topological descriptors such as the number of atoms of each kind and the number of chemical bonds. The set is constructed by taking the linear, quadratic, and cubic potencies of the following descriptors: {C, H, C-C, C=C, $C\text{-}C_{triple}$, $C\text{-}C_{aromatic}$, $C_{primary}$, $C_{secondary}$, $C_{tertiary}$, $C_{quaternary}$}, then leading to a number of 30 descriptors.

If we study atoms and bonds separately, it is not possible to obtain a good model. Applying Maronna´s method, we got a model with 11 descriptors that perform better than the previous ones.

**Model 2**

$$DM = –0.1961C – 0.1010H + 0.1674C\text{-}C + 0.5496\, C_{primary} + 0.0464[C]^2 –0.0889.[C{=}C]^2 – \\ –0.0105[C\text{-}C_{aromatic}]^2 – 0.1849[C_{primary}]^2 – 0.0024[C]^3 + 0.0137[C{=}C]^3 + \\ + 0.0008[C\text{-}C_{aromatic}]^3 + 0.8309$$

$R_{cal} = 0.9486$, $S_{cal} = 0.0808$, $F = 18.8$, absdv = 0.0496, $R_{lou} = 0.9063$, $S_{lou} = 0.0884$

There are no-outliers with absolute deviation exceeding 2.5*S. Model 2 clearly outperforms results from model 1 and the reported one using a flexible set of molecular descriptors[19]. The predicted values of DM are given in Table 1.

A further step in modeling the DM was to combine the different sets discussed above in a unique set. Joining the conventional topological indices, the $S_i$'s, and atoms and bonds, we were not able to obtain a model better than model 2. In fact, we found that the best one-descriptor model of the total set is given by the descriptor $C\text{-}C_{triple}$, and that the best two-descriptor model includes the descriptor $C\text{-}C_{aromatic}$, thus suggesting the relative importance of the bond descriptors for the property. However, this conclusion is dependent on the dimension of the molecular set used.

The DERIVE programs used in this work are available on request to one of us (PRD).

## CONCLUSIONS

The simplest and most elementary set of topological descriptors, the number of atoms of each kind and the number of chemical bonds, give better results for modeling the molecular charge distribution than the more elaborated topological indices, including those that can explain some electronic effects such as the $S_i$'s.

This result can be easily understood. It is a well-known fact that a statistical study does not allow one to derive unambiguous physical chemistry interpretations on the basis of the molecular descriptors employed to derive a model. In other words, there are no universal rules to draw conclusions on the basis of a particular set of molecular or/and topological descriptors because a relatively good predictive power of the model does not lead to cause-effect relationships between property and independent variables. Thus, the naïveté of these descriptors does not lead to a poorer model than that obtained with more sophisticated indices.

One must take into account the extremely simple computational procedure to derive the basic descriptors: just to count and to identify atoms and chemical bonds. Although the employment of these basic molecular descriptors does not provide any physical insight into the molecular behavior, they make clear the fundamental value of the atom and bond concepts within the framework of the molecular structure paradigm. After all, other more elaborated molecular descriptors always contain some degree of arbitrariness. This is an unavoidable fact due to the strong reduction process when trying to describe a so complex and rather subtle issue as it is the molecular structure concept via a single figure.

The conclusions obtained in this paper are in line with other similar findings reported before[42,43,44,45]. At present, we are studying other molecules and different activities and properties to support the motivating idea of this work. Results will be given elsewhere in the near future.

## ACKNOWLEDGMENTS

We wish to thank Dr. Ricardo Maronna of the Mathematics Department of Facultad de Ciencias Exactas, Universidad Nacional de La Plata for providing us with valuable and significative statistical ideas. We have also to recognize the valuable comments of two anonymous referees that have been useful to improve the final version of this article.
